# SECONDARY EMOTIONAL DISTRESS: SUPPORTING YOUR RESEARCH TEAM THROUGH THE QUALITATIVE RESEARCH PROCESS

**DOI:** 10.1093/geroni/igae098.3185

**Published:** 2024-12-31

**Authors:** Kathryn Coccia, Verna Hendricks-Ferguson, Stephanie Wladkowski, Cara Wallace

**Affiliations:** Saint Louis University, St. Louis, Missouri, United States; Saint Louis University, St. Louis, Missouri, United States; Bowling Green State University, College of Health & Human Services, Bowling Green, Ohio, United States; Saint Louis University, St. Louis, Missouri, United States

## Abstract

Conducting qualitative interviews with older adults experiencing serious illness as a patient or caregiver may include distressing subject matter for researchers. A growing body of evidence shows researchers are at risk of developing secondary emotional distress from eliciting or processing participants’ distressing experiences. Novice researchers or graduate research assistants may be particularly vulnerable to developing secondary emotional distress. Many qualitative research plans include protocols for managing participant distress, but very few include formal considerations for mitigating psychological risk for members of the research team. Using a case study from an ongoing NIH-funded research study on live discharge from hospice, this presentation will focus a wider discussion of how difficult emotional experiences can occur both during the data collection process and again in analysis. This phenomenon will be discussed from the lens of a graduate research assistant, the primary investigator, and senior co-investigators. Presenters will share how these situations were processed, insights gleaned from these experiences, and considerations for developing protocols and cultivating resources to support team members in future studies.

